# Structural basis for backtracking by the SARS-CoV-2 replication–transcription complex

**DOI:** 10.1073/pnas.2102516118

**Published:** 2021-04-21

**Authors:** Brandon Malone, James Chen, Qi Wang, Eliza Llewellyn, Young Joo Choi, Paul Dominic B. Olinares, Xinyun Cao, Carolina Hernandez, Edward T. Eng, Brian T. Chait, David E. Shaw, Robert Landick, Seth A. Darst, Elizabeth A. Campbell

**Affiliations:** ^a^Laboratory of Molecular Biophysics, The Rockefeller University, New York, NY 10065;; ^b^D. E. Shaw Research, New York, NY 10036;; ^c^Laboratory of Mass Spectrometry and Gaseous Ion Chemistry, The Rockefeller University, New York, NY, 10065;; ^d^Department of Biochemistry, University of Wisconsin–Madison, Madison, WI 53706;; ^e^The National Resource for Automated Molecular Microscopy, Simons Electron Microscopy Center, New York Structural Biology Center, New York, NY 10027;; ^f^Department of Biochemistry and Molecular Biophysics, Columbia University, New York, NY 10032;; ^g^Department of Bacteriology, University of Wisconsin–Madison, Madison, WI 53706

**Keywords:** coronavirus, backtracking, cryo-electron microscopy, molecular dynamics, RNA-dependent RNA polymerase

## Abstract

Backtracking, the reverse motion of the transcriptase enzyme on the nucleic acid template, is a universal regulatory feature of transcription in cellular organisms but its role in viruses is not established. Here we present evidence that backtracking extends into the viral realm, where backtracking by the severe acute respiratory syndrome coronavirus 2 (SARS-CoV-2) RNA-dependent RNA polymerase (RdRp) may aid viral transcription and replication. Structures of SARS-CoV-2 RdRp bound to the essential nsp13 helicase and RNA suggested the helicase facilitates backtracking. We use cryo-electron microscopy, RNA–protein cross-linking, and unbiased molecular dynamics simulations to characterize SARS-CoV-2 RdRp backtracking. The results establish that the single-stranded 3′ segment of the product RNA generated by backtracking extrudes through the RdRp nucleoside triphosphate (NTP) entry tunnel, that a mismatched nucleotide at the product RNA 3′ end frays and enters the NTP entry tunnel to initiate backtracking, and that nsp13 stimulates RdRp backtracking. Backtracking may aid proofreading, a crucial process for SARS-CoV-2 resistance against antivirals.

## Significance

The COVID-19 pandemic is caused by the severe acute respiratory syndrome coronavirus 2 (SARS-CoV-2). The SARS-CoV-2 genome is replicated and transcribed by its RNA-dependent RNA polymerase (RdRp), which is the target for antivirals such as remdesivir. We use a combination of approaches to show that backtracking (backward motion of the RdRp on the template RNA) is a feature of SARS-CoV-2 replication/transcription. Backtracking may play a critical role in proofreading, a crucial process for SARS-CoV-2 resistance against many antivirals.

Severe acute respiratory syndrome coronavirus 2 (SARS-CoV-2) is the causative agent of the current COVID-19 pandemic (1, 2). The SARS-CoV-2 genome is replicated and transcribed by its RNA-dependent RNA polymerase holoenzyme [holo-RdRp, subunit composition nsp7/nsp8_2_/nsp12 (3, 4)] in a replication–transcription complex (RTC), which is the target for antivirals such as remdesivir (Rdv) (5). The holo-RdRp is thought to coordinate with many cofactors to carry out its function (6, 7). Some of these cofactors, such as the nsp13 helicase (8) and the nsp10/nsp14 proofreading assembly (9, 10), are also essential for viral replication and are antiviral targets (11–13).

We recently reported views of the SARS-CoV-2 RTC in complex with the nsp13 helicase [cryo-electron microscopy (cryo-EM) structures at a nominal resolution of 3.5 Å (14)]. The overall architecture of the nsp13-RTC places the nucleic acid binding site of nsp13 directly in the path of the downstream template-strand RNA (t-RNA), and cryo-EM difference maps revealed the 5′-single-stranded t-RNA overhang engaged with nsp13 before entering the RdRp active site (14). The nsp13 helicase translocates on single-stranded nucleic acid in the 5′→3′ direction (15–22). Thus, this structural arrangement presents a conundrum: The RdRp translocates in the 3′→5′ direction on the t-RNA strand, while nsp13 translocates on the same strand in the opposite direction. Translocation of each enzyme opposes each other, and if the helicase prevails it is expected to push the RdRp backward on the t-RNA (14). This reversible backward sliding, termed backtracking, is a well-studied feature of the cellular DNA-dependent RNA polymerases (DdRps) (23–30).

Backtracking by the cellular DdRps plays important roles in transcription regulation, including the control of DdRp pausing during transcription elongation, termination, DNA repair, and transcription fidelity (25). In backtracking, the DdRp and associated transcription bubble move backward on the DNA, while the RNA transcript reverse-threads through the complex to maintain the register of the RNA–DNA hybrid (23–30). This movement generates a single-stranded 3′ segment of the RNA transcript which is extruded out the secondary or nucleoside triphosphate (NTP) entry tunnel that branches off from the primary DdRp active-site cleft around the conserved bridge helix (27–31).

Although evolutionarily unrelated to the DdRps, a secondary channel, formed by the RdRp motif F β-hairpin loop and proposed to serve as an NTP entry tunnel, branches off from the main SARS-CoV-2 RdRp active-site channel (32). This NTP entry tunnel is well positioned to receive the single-stranded 3′ segment of backtracked RNA, a structural architecture analogous to the DdRps (14). We envisaged that translocation by the helicase could mediate backtracking of the RdRp, an otherwise energetically unfavorable process, enabling the key viral functions such as proofreading (9, 10, 12, 33) and template switching during subgenomic transcription (7, 34). Here we outline the structural basis for SARS-CoV-2 RTC backtracking and describe the role of nsp13 in stimulating backtracking.

## Results

### SARS-CoV-2 RdRp Backtracked Complexes for Cryo-EM.

**Fig. 1.**
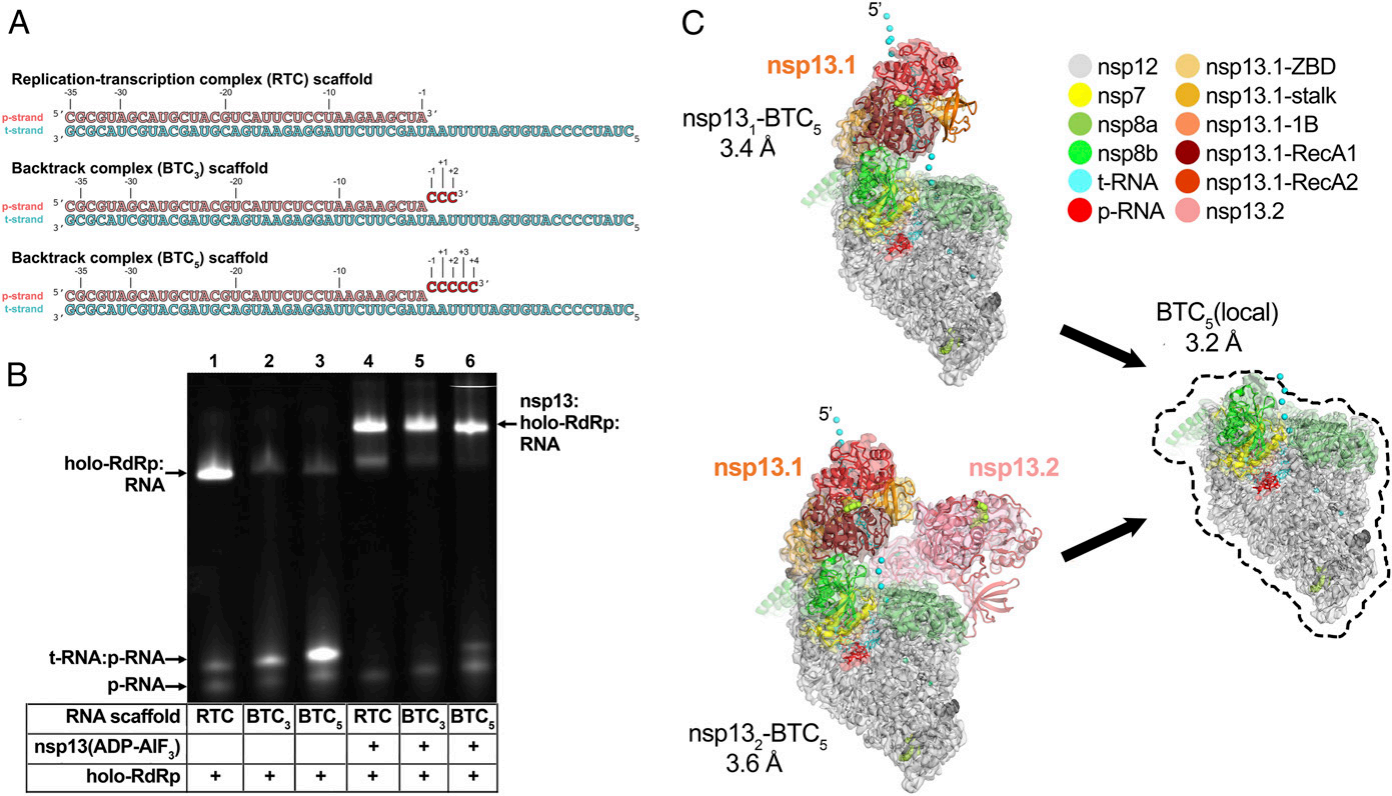
SARS-CoV-2 backtrack complex. (*A*) RNA scaffolds: (*Top*) RTC scaffold (14); (*Bottom*) backtrack complex scaffolds (BTC_3_ and BTC_5_). (*B*) A native gel electrophoretic mobility shift assay reveals that holo-RdRp requires nsp13(ADP-AlF_3_) to bind the BTC scaffolds efficiently. (*C*) Cryo-EM structures of SARS-CoV-2 BTCs. Shown is the transparent cryo-EM density [local-resolution-filtered (47)] with the refined models superimposed (*SI Appendix*, Table S1). The models and density are colored according to the key. Two major BTCs were observed (*SI Appendix*, Fig. S2), one containing one nsp13 protomer (nsp13_1_-BTC_5_), and one containing two nsp13 promoters (nsp13_2_-BTC_5_). We designate the nsp13 promoter common to both structures nsp13.1 and the other nsp13.2 (14). The cyan spheres denote the path of the single-stranded t-RNA 5′ segment, some of which is engaged with nsp13.1 in both structures.

Previously, DdRp backtracked complexes (BTCs) were generated for structural studies by direct incubation of the DdRp with DNA–RNA scaffolds containing mismatched nucleotides at the RNA 3′ end (27, 28, 30); these BTC scaffolds bind with the downstream Watson–Crick base pairs of the RNA–DNA hybrid positioned in the DdRp active site and the single-stranded 3′ segment of mismatched RNA extruding out the DdRp NTP entry tunnel. To study RdRp BTCs, we therefore designed and tested RNA scaffolds based on our original SARS-CoV-2 RTC scaffold but with three or five mismatched cytosine nucleotides added to the product RNA (p-RNA) 3′ end (BTC_3_ and BTC_5_ scaffolds; Fig. 1*A*). Consecutive mismatches at the p-RNA 3′ end were designed to generate stable, homogeneous BTCs for biochemical and structural analysis—we do not propose that consecutive mismatches are biologically relevant.

Native electrophoretic mobility shift assays revealed that although the holo-RdRp (nsp7/nsp8_2_/nsp12) bound the RTC scaffold as observed previously (Fig. 1*B*, lane 1, *SI Appendix*, Fig. S1*A*, and ref. 14), nsp13 was required for efficient binding to the BTC scaffolds (Fig. 1*B*). Stable nsp13–holo-RdRp complexes with BTC scaffolds were also observed by native mass spectrometry (*SI Appendix*, Fig. S1 *B* and *C*).

Modeling suggested that about five nucleotides of backtracked single-stranded RNA at the p-RNA 3′ end would be sufficient to traverse the RdRp NTP entry tunnel. Therefore, to determine the structural organization of the SARS-CoV-2 BTC, we assembled nsp13(ADP-AlF_3_) and holo-RdRp with the BTC_5_ scaffold (Fig. 1*A*; hereafter called BTC_5_) and analyzed the samples by single-particle cryo-EM. The sample comprised two major classes: nsp13_1_-BTC_5_ (3.4-Å nominal resolution) and nsp13_2_-BTC_5_ (3.6 Å; Fig. 1*C* and *SI Appendix*, Figs. S2 and S3). Analysis of the two refined structures revealed that the RdRp portion of each structure was essentially identical (rmsd of 927 nsp12 α-carbon positions <0.3 Å; *SI Appendix*, Table S2), while the disposition of the common nsp13 protomer (nsp13.1) was divergent (rmsd of 590 nsp13 α-carbon positions >8 Å; *SI Appendix*, Table S2). To eliminate structural heterogeneity in the nsp13 subunits and obtain a higher-resolution view of the BTC, the particles from both classes were combined and locally refined inside a mask applied around the holo-RdRp and RNA (excluding the nsp13 subunits), leading to the BTC_5_(local) combined map (3.2 Å; Fig. 1*C* and *SI Appendix*, Figs. S2 and S3 and Table S1).

**Fig. 2.**
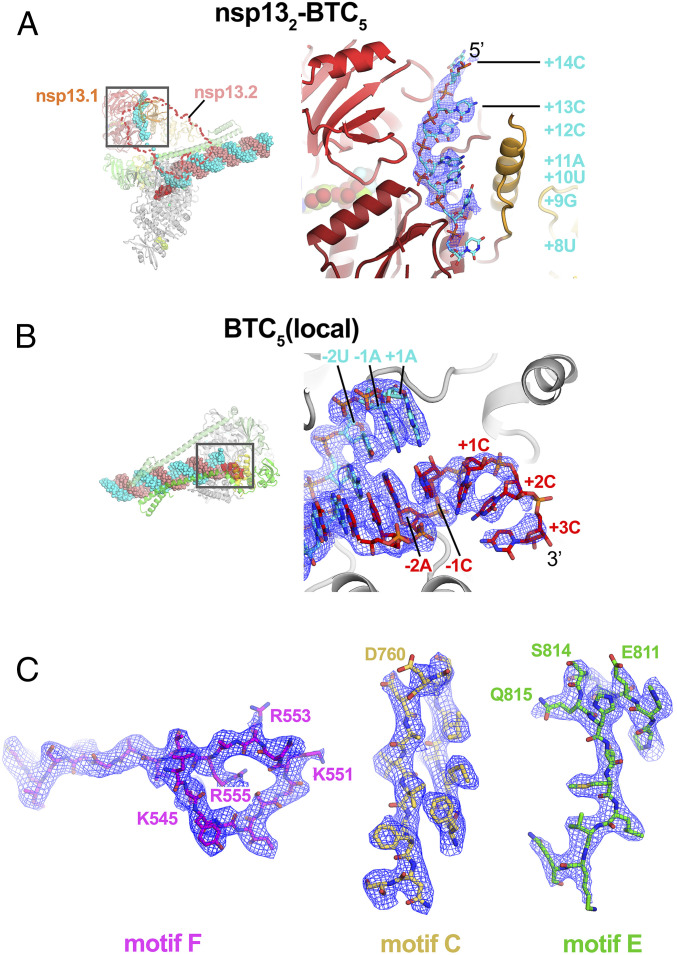
Cryo-EM density maps. (*A*, *Left*) Overall view of nsp13_2_-BTC_5_. Nsp13.2 is removed (outline) for clarity. The boxed region is magnified on the right. (*A*, *Right*) Magnified view of the t-RNA segment (+14-5′-CCCAUGU-3′-+8) enclosed in the nsp13.1 helicase subunit. The cryo-EM density map (from the nsp13_2_-BTC structure) for the RNA is shown (blue mesh). (*B*, *Left*) Overall view of the BTC_5_(local) structure. The boxed region is magnified on the right. (*B*, *Right*) Magnified view of the region around the RdRp active site, showing the t-RNA (cyan) and p-RNA (red) with the backtracked RNA segment. The cryo-EM density map for the RNA [from BTC_5_(local)] is shown (blue mesh). (*C*) BTC_5_(local) cryo-EM density maps around nsp12 conserved motifs F, C, and E. Selected residues are labeled.

The cryo-EM maps (Figs. 1*C* and 2) revealed two significant differences with the nsp13-RTC structures (14): 1) The single-stranded downstream template RNA (t-RNA) engaged with nsp13.1 was resolved (Fig. 2*A*), and 2) a single-stranded p-RNA 3′ segment was extruded into the RdRp NTP entry tunnel (Fig. 2*B*).

### Nsp13 Binds the Downstream Single-Stranded t-RNA.

In the nsp13_1_-BTC_5_ and nsp13_2_-BTC_5_ cryo-EM maps, the single-stranded 5′ segment of the t-RNA was engaged with nsp13.1. This region of the cryo-EM density was well-resolved (Fig. 2*A*), allowing identification of the t-RNA segment engaged within the helicase as +14 to +8 (numbering defined in Fig. 1*A*), ^5′^CCCAUGU^3′^. The five-nucleotide segment connecting the t-RNA between the helicase and the RdRp (+7 to +3) was disordered and not modeled.

### The SARS-CoV-2 RdRp NTP Entry Tunnel Accommodates the Backtracked RNA.

**Fig. 3.**
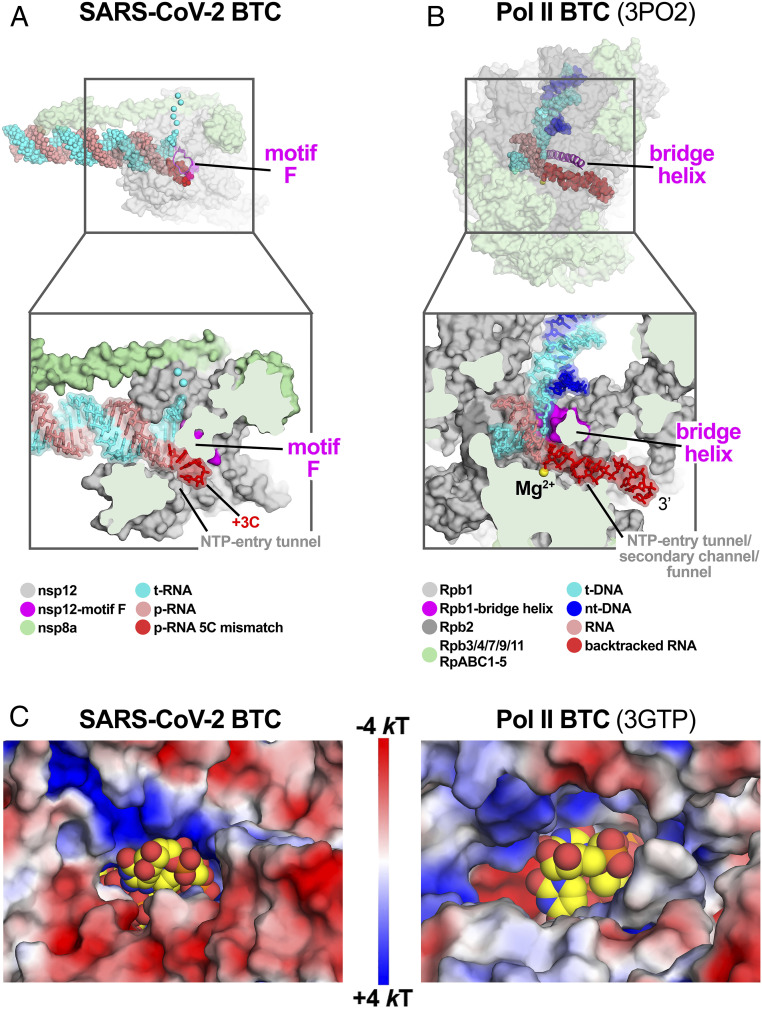
SARS-CoV-2 RdRp and DdRp BTCs. (*A* and *B*) SARS-CoV-2 RdRp (*A*) and DdRp (*B*) BTCs. (*Top*) Proteins are shown as transparent molecular surfaces and nucleic acids as atomic spheres. The boxed regions are magnified on the bottom. (*Bottom*) Magnified, cross-sectional view. Proteins are shown as molecular surfaces and nucleic acids in stick format with transparent molecular surface. (*A*) The SARS-CoV-2 BTC_5_(local). Nsp8a and nsp12 are shown (nsp7 and nsp8b are removed for clarity). Nsp12 motif F is shown as a magenta backbone ribbon (*Top*). Backtracked RNA (+1C to +3C of the BTC_5_-scaffold; Fig. 1*A*) extrudes out the NTP entry tunnel. (*B*) A DdRp (*Saccharomyces cerevisiae* Pol II) BTC [Protein Data Bank (PDB) ID code 3PO2 (29)]. The BH is shown as a magenta backbone ribbon. The backtracked RNA extrudes out the NTP entry tunnel/secondary channel/funnel. (*C*) Views from the outside into the NTP entry tunnels of the SARS-CoV-2 (*Left*) and an *S. cerevisiae* DdRp [PDB ID code 3GTP (27)] BTC. Protein surfaces are colored by the electrostatic surface potential [calculated using APBS (48)]. Backtracked RNA is shown as atomic spheres with yellow carbon atoms.

The cryo-EM maps also resolved a single-stranded p-RNA 3′ segment of the BTC_5_ scaffold extruding into the RdRp NTP entry tunnel (Fig. 2*B*), confirming the formation of a BTC (Fig. 3*A*). The overall architecture of the SARS-CoV-2 BTC is analogous to DdRp BTCs (Fig. 3 and ref. 14). The DdRp bridge helix (BH) (35) separates the DdRp active site cleft into a channel for the downstream template DNA (over the top of the BH; Fig. 3*B*) and the NTP entry tunnel (underneath the BH; Fig. 3*B*). Similarly, the viral RdRp motif F (*SI Appendix*, Fig. S4*A* and ref. 32) serves as the strand-separating structural element for the backtracked RNA (Fig. 3*A*). The downstream t-RNA passes over the top of motif F, while the backtracked RNA extrudes out the NTP entry tunnel underneath motif F (Fig. 3*A*).

**Fig. 4.**
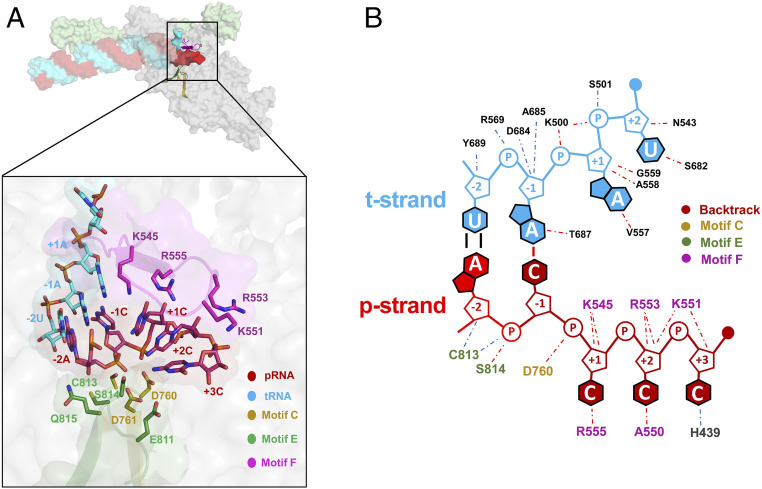
Protein–RNA interactions in the BTC. (*A*, *Top*) Overall view of BTC_5_(local). Proteins are shown as transparent molecular surfaces and nucleic acids as atomic spheres. Nsp8a and nsp12 are shown (nsp7 and nsp8b are removed for clarity). Nsp12 motifs C, E, and F are shown as backbone ribbons (colored according to the key on the bottom). The boxed region is magnified below. (*A*, *Bottom*) RNA is shown from −2 to +3. Proteins are shown as transparent molecular surfaces. RdRp motifs C, E, and F are shown as transparent backbone ribbons (colored according to the key) with side chains of residues that approach the backtracked RNA (≤4.5 Å) shown. (*B*) Schematic illustrating the same protein–RNA interactions as *A*. Drawn using Nucplot (49).

The RdRp NTP entry tunnel provides a steric and electrostatic environment conducive to channeling the backtracked RNA out of the active site without specific polar protein–RNA interactions that could hinder the RNA movement (Figs. 3*C* and 4). Comparing the electrostatic surface potential of the NTP entry tunnels of the SARS-CoV-2 RdRp with eukaryotic and bacterial DdRps reveals a similar overall electrostatic surface environment that may facilitate backtracked RNA entry (Fig. 3*C* and *SI Appendix*, Fig. S4*B*), including a “track” of conserved positively charged Arg and Lys residues of motif F (SARS-CoV-2 nsp12 K545, K551, R553, and R555; Fig. 4 and *SI Appendix*, Fig. S4*A*). Conserved residues of RdRp motifs C and E complete the active-site/NTP entry tunnel environment surrounding the backtracked RNA (Fig. 4 and *SI Appendix*, Fig. S4*A*).

**Fig. 5.**
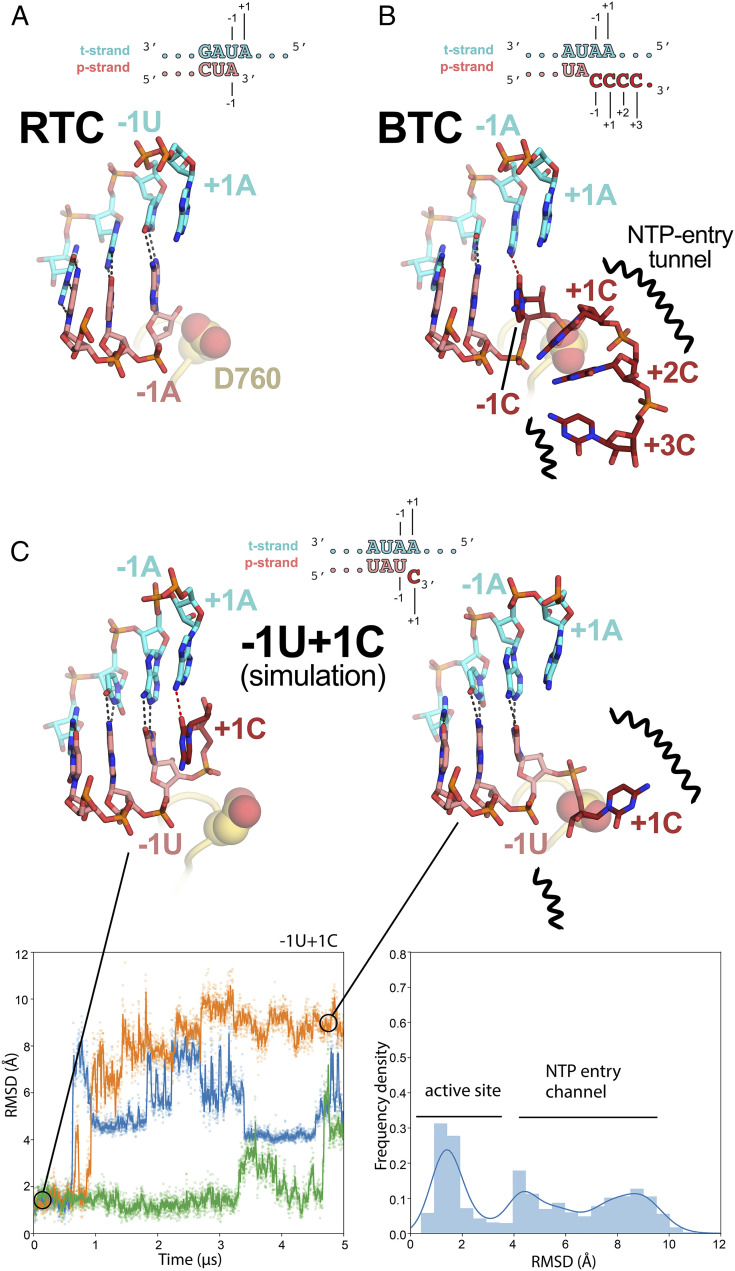
Comparison of active-site proximal RNA in the RTC and BTC structures and from simulations of a mismatched nucleotide at the p-RNA 3′ end. (*A* and *B*) Comparison of the active-site proximal RNA in the RTC [*A*; PDB ID code 6XEZ (14)], BTC_5_(local) (*B*), and from selected snapshots of molecular dynamics simulations of a −1U + 1C complex (*C*). The schematics denote the nucleotides shown in the context of the RTC (*A*) and BTC_5_ scaffolds (*B*; full scaffold sequences shown in Fig. 1*A*) or generated from the BTC_5_ scaffold for the simulations (*C*). Carbon atoms of the t-RNA are colored cyan and p-RNA are colored salmon except in the case of mismatched Cs at the 3′ end, which are colored dark red. Watson–Crick base-pairing hydrogen bonds are denoted as dark gray dashed lines; other hydrogen-bonds as red dashed lines. Nsp12 motif C is shown as a yellow-orange backbone ribbon, and the side chain of D760 is shown as atomic spheres. (*A*) The RTC is in a posttranslocated state, with the A–U base pair at the p-RNA 3′ end in the −1 position (14). (*B*) The BTC_5_(local) RNA is translocated compared to the RTC; the base pair corresponding to A–U at the 3′ end of the RTC RNA in the −1 position is in the −2 position of the BTC RNA. A C–A mismatch occupies the BTC −1 site. The +1, +2, and +3 mismatched Cs trail into the RdRp NTP entry tunnel (denoted by black squiggly lines). The +4C (present in the BTC_5_ scaffold; Fig. 1*A*) is exposed to solvent, disordered, and not modeled. (*C*) Molecular dynamics simulations of the nsp13_2_–BTC_−1U+1C_ complex. The complex was simulated with three replicates (green, blue, and orange traces). Rmsd values plotted as a function of time represent the heavy-atom rmsd of the +1C of the p-RNA compared with the starting configuration (*Materials and Methods*). The rmsd histograms (plotted on the right) are an aggregate of all three replicates. Two structures taken from one of the simulations are shown, one showing the +1C of the p-RNA in the active site (*t* = 0 μs) and the other showing the +1C frayed into the NTP entry tunnel (*t* = 4.5 μs).

In the nsp13-RTCs, the RTC scaffold (Fig. 1*A*) is bound in a posttranslocated state (14); the 3′ p-RNA A is base-paired to the t-RNA U at the −1 site near the catalytic nsp12-D760 (Fig. 5*A*). The next t-RNA base (A at +1) is positioned to receive the incoming NTP substrate, but the site for the incoming NTP substrate is empty (Fig. 5*A*). By contrast, the BTC structures were translocated by one base pair compared to the RTCs; the base pair corresponding to the A–U Watson–Crick base pair at the 3′ end of the p-RNA (located in the −1 site of the RTCs) was in the −2 position of the BTCs (Figs. 1*A*, 4, and 5*B*). The −1 position of the BTC was occupied by the first C–A mismatch; the p-RNA −1C made a non-Watson–Crick hydrogen bond with the opposing t-RNA A (Figs. 4 and 5*B*). The next three mismatched p-RNA nucleotides (+1C, +2C, and +3C) trailed into the NTP entry tunnel (Figs. 4 and 5*B*). The 3′ nucleotide of the BTC_5_ scaffold p-RNA (+4C; Fig. 1*A*) was solvent-exposed at the outward-facing end of the NTP entry tunnel and lacked density and was therefore not modeled (Fig. 2*B*). The trajectory of the backtracked nucleotides at positions +1/+2 was sharply bent due to spatial constraints of motif F residues (Fig. 4*A*).

### Nsp13 Stimulates Backtracking.

The SARS-CoV-2 wild-type holo-RdRp required the nsp13 helicase to bind the BTC scaffolds efficiently (Fig. 1*B*). However, we observed that the holo-RdRp containing nsp12 with a single amino acid substitution (D760A) did not require nsp13 to bind the BTC scaffolds (*SI Appendix*, Fig. S1*A*, lane 4). Nsp12-D760 is a conserved residue of the RdRp motif C that chelates a crucial Mg^2+^ ion in catalytic complexes (*SI Appendix*, Fig. S4*A* and ref. 32), but in RdRp structures lacking substrate (including the BTC structures) the Mg^2+^ ions are absent (14, 36, 37). The catalytic Asp residues of the DdRps typically chelate the Mg^2+^ ion even in the absence of substrate (31, 38), and this Mg^2+^ is retained in DdRp backtracked structures (27–30). Our RdRp BTC structures suggest that in the absence of a Mg^2+^ ion D760 presents an electrostatic barrier to the phosphate backbone of the backtracked RNA (Fig. 5*B*), explaining the requirement for the helicase to surmount this barrier and why removal of D760 stabilizes binding to the BTC scaffolds.

To generate the SARS-CoV-2 BTCs for structural studies, we used the BTC_5_ scaffold with five mismatched Cs at the p-RNA 3′ end (Fig. 1*A*). To study the formation of SARS-CoV-2 BTCs from an RTC scaffold (fully Watson–Crick base-paired p-RNA 3′ end), we analyzed ultraviolet (UV)-induced cross-linking from 4-thio-U incorporated penultimate to the p-RNA 3′ end [RTC(4-thio-U)-scaffold; *SI Appendix*, Fig. S5*A* and ref. 39]. Cross-linking was absolutely dependent on the presence of 4-thio-U in the RNA, establishing specificity (*SI Appendix*, Fig. S5*B*). RTCs assembled with wild-type nsp12 and the RTC(4-thio-U) scaffold gave weak nsp12-RNA cross-linking upon UV exposure (*SI Appendix*, Fig. S5*A*, lane 1). These conditions favor a posttranslocated RTC (14, 36, 37) with the 4-thio-U sequestered in the RNA–RNA hybrid and thus not available for protein–RNA cross-linking. Cross-linking of the p-RNA to nsp12 was substantially increased by the addition of nsp13 with 2 mM adenosine 5′-triphosphate (ATP) (*SI Appendix*, Fig. S5*A*, lane 2). Under these conditions, we propose that the translocation activity of nsp13 backtracked a fraction of the complexes, freeing the 4-thio-U from the RNA–RNA hybrid for cross-linking to nsp12. Cross-linking in the presence of nsp13 but in the absence of ATP reduced nsp12 cross-linking (*SI Appendix*, Fig. S5*A*, lane 7 versus lane 2), supporting the proposal that nsp13 translocation activity facilitates backtracking. Replacing wild-type nsp12 with nsp12-D760A (nsp12*; *SI Appendix*, Fig. S5*A*, lanes 4 to 6, 9, and 10), which is more prone to backtracking (*SI Appendix*, Fig. S1*A*), showed the same trends but with increased UV-dependent nsp12-RNA cross-linking, with the maximal cross-linking occurring under the conditions expected to favor backtracking the most (*SI Appendix*, Fig. S5*A*, lane 5). These results affirm the view that nsp13 facilitates backtracking of the SARS-CoV-2 RdRp.

### A Mismatched Nucleotide at the p-RNA 3′ End Spontaneously Frays and Enters into the RdRp NTP Entry Tunnel.

The SARS-CoV-2 RTC is a highly processive and rapid replicase/transcriptase, capable of replicating a ∼1-kb RNA template at an average rate of ∼170 nt/s (40). However, studies of other viral RdRps suggest that misincorporation slows the overall elongation rate and may induce backtracking (41–43). We used molecular dynamics simulations to explore the fate of a mismatched nucleotide incorporated at the p-RNA 3′ end. Starting with the nsp13_2_-BTC_5_ structure, the −1C was mutated to U, and the +2 to +4 Cs were removed. The resulting pretranslocated p-RNA had a matched −1U and a mismatched +1C (−1U + 1C; Fig. 5*C*). In three 5-μs simulations we observed the 3′-mismatched +1C alternating between two positions, either remaining in the vicinity of the active site (rmsd <3.5 Å) or fraying away from the p-RNA:t-RNA hybrid toward or into the NTP entry tunnel (rmsd >3.5 Å; Fig. 5*C*). Based on analysis of the aggregated −1U + 1C simulations, the mismatched +1C spent about 40% of the time near the active site and about 60% of the time frayed toward or in the NTP entry tunnel. In control simulations with a fully matched p-RNA 3′ end (−1U + 1U), the matched +1U at the p-RNA 3′ end did not fray and spent 100% of the time in the active-site pocket (*SI Appendix*, Fig. S6).

Nucleotides −36 to +14 of the BTC_5_ scaffold t-RNA (as defined in Fig. 1*A*) were included in the simulations. The nsp13.1-bound (+8 to +14) and the nsp12-bound (−36 to +2) regions were stable over the course of the simulation time. The t-RNA nucleotides +3 to +7 (the portion connecting the nsp12-bound and nsp13.1-bound t-RNA) were highly dynamic, consistent with the absence of well-defined cryo-EM density for this region of the t-RNA. We note that the simulations inform on the path of frayed RNAs but not on the role of nsp13 in backtracking.

## Discussion

**Fig. 6.**
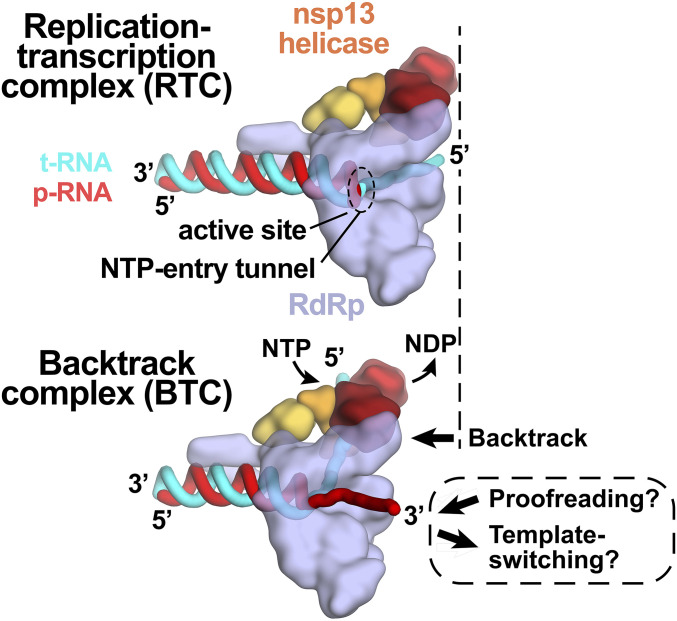
Role of backtracking in proofreading and template switching during subgenomic transcription. Schematic illustrating the proposed model for backtracking of the SARS-CoV-2 RTC and its potential role in proofreading and template switching during subgenomic transcription. The structural models are shown as cartoons (holo-RdRp, light blue; nsp13 helicase, orange shades; RNA strands, colored tubes as indicated). (*Top*) In the RTC, the elongating RdRp moves from left to right. The RdRp active site holds the p-RNA 3′ end. The NTP entry tunnel provides access from solution to the RdRp active site. The downstream (5′) single-stranded t-RNA is not engaged with nsp13. (*Bottom*) In the BTC, nsp13 translocates on the downstream (5′) single-stranded t-RNA, pushing the RdRp backward (right to left) on the RNA. This causes the p-RNA to reverse-thread through the complex, with the resulting single-stranded 3′ fragment extruding out the NTP entry tunnel. The exposure of the p-RNA 3′ end could facilitate proofreading (9, 10, 12, 50) and also template switching during subgenomic transcription (7, 34).

Our results establish that the SARS-CoV-2 RTC backtracks, that backtracking is facilitated by the nsp13 helicase, and that the resulting single-stranded 3′ segment of the p-RNA extrudes out the RdRp NTP entry tunnel in a manner analogous to the evolutionarily unrelated cellular DdRps (Fig. 3). Thus, a secondary tunnel to accommodate backtracked RNA, facilitating fidelity and possibly other functions (Fig. 6), appears to be a crucial feature of transcriptase enzymes that evolved independently.

Backtracking of Φ6 and poliovirus RdRps has been reported based on analysis of single-molecule observations (41–43). The nsp13 helicase facilitates efficient backtracking of the SARS-CoV-2 RTC (*SI Appendix*, Fig. S5). We note that in bacteria the UvrD helicase has been shown to induce DdRp backtracking, suggesting that a role for helicases in backtracking may be widespread (44). Here we envision the helicase translocating on the downstream t-RNA, facilitating unwinding of the duplex t-RNA/p-RNA and entry of the p-RNA 3′-single-stranded fragment into the NTP entry tunnel. This process could be triggered by a mismatched nucleotide at the p-RNA 3′ end.

Our results are consistent with the view that a matched nucleotide at the pretranslocated p-RNA 3′ end remains base paired to the t-RNA (Fig. 5 and *SI Appendix*, Fig. S6), facilitating translocation and subsequent NTP addition and thus rapid elongation (at a maximum elongation rate of ∼170 nt/s a translocation event would occur approximately every 6 ms, on average, explaining why translocation was not observed in our 5-μs simulations; Fig. 5 and *SI Appendix*, Fig. S6). However, upon misincorporation, the pretranslocated, mismatched nucleotide at the p-RNA 3′ end spends more than half the time frayed from the t-RNA and toward or in the NTP entry tunnel (Fig. 5*C*), a configuration that is likely recalcitrant to translocation and subsequent elongation. The favorable environment of the NTP entry tunnel (Figs. 3 and 4) may further encourage backtracking. The resulting inhibition of translocation may enable the tight engagement of the nsp13.1 helicase with the downstream single-stranded t-RNA (Fig. 2*A*), allowing the 5′→3′ translocation activity of the helicase to more robustly backtrack the complex (*SI Appendix*, Fig. S5).

Our findings have implications for the processes of subgenomic transcription and proofreading in SARS-CoV-2 (Fig. 6 and ref. 14). Generation of messenger RNAs for the viral structural proteins begins with transcription initiation at the 3′-poly(A) tail of the (+)-strand RNA genome. The process of subgenomic transcription ultimately generates a nested set of transcripts that are both 5′- and 3′-coterminal with the viral genome and involves a remarkable template switch from the 3′ portion of the genome to the 5′ leader (7, 34). The template-switching event is thought to involve stalling of the RdRp then base-pairing between the 3′ end of the nascent transcript and a complementary sequence (the transcription regulatory sequence, or TRS) near the (+)-strand 5′ leader (45). The 3′ end of the nascent transcript is base-paired to the t-RNA and is sequestered in the stalled RdRp active site; for template switching to occur the 3′ end of the nascent transcript must be separated from the t-RNA and from the RdRp active site so that it is available for base pairing to the TRS near the 5′ leader. Backtracking would separate the p-RNA 3′ end from the t-RNA and would also extrude the 3′ end of the nascent transcript out the NTP entry tunnel, making it available for base pairing to the 5′ TRS (Fig. 6). Our results establishing that the SARS-CoV-2 RTC can backtrack validates a key prediction of this model for the mechanism of template switching during subgenomic transcription (14).

Nucleotide analogs that function by being incorporated into product RNA by viral RdRps are important antiviral therapeutics (46). Notably, their incorporation may induce backtracking by the RdRp (43). Rdv, a nucleotide analog, is the only Food and Drug Administration–approved drug for COVID-19 treatment (5). Our results support a model in which RdRp misincorporation or incorporation of nucleotide analogs can pause the RdRp, allowing nsp13 to engage with the downstream single-stranded t-RNA to induce backtracking (14). The resulting exposure of the p-RNA 3′ end out the NTP entry tunnel (Figs. 3*A* and 6) could provide access for the SARS-CoV-2 proofreading machinery (nsp10/14) (9, 12) to degrade the p-RNA 3′ end, thus removing the misincorporation or analog. This proofreading activity, which is unique to the nidovirus order to which CoVs belong (10), is a major determinant for the resistance of CoVs against many nucleotide analog inhibitors (13). Thus, understanding RdRp backtracking and its potential role in CoV proofreading can facilitate the development of therapeutics.

## Materials and Methods

Detailed descriptions of SARS-CoV-2 nsp12, 7, 8, and 13 protein purification, assembly of the RTC complexes, native electrophoretic mobility shift assays, native mass spectrometry, cross-linking, specimen preparation for cryo-EM, cryo-EM data acquisition and processing, model building and refinement, and molecular dynamics simulations are provided in *SI Appendix*.

## Supplementary Material

Supplementary File

Supplementary File

## Data Availability

The cryo-EM density maps have been deposited in the EMDataBank: EMD-23007 (nsp13_1_-BTC_5_), EMD-23008 (nsp13_2_-BTC_5_), and EMD-23009 [BTC_5_(local)]. Atomic coordinates have been deposited in the Protein Data Bank: 7KRN (nsp13_1_-BTC_5_), 7KRO (nsp13_2_-BTC_5_), and 7KRP [BTC_5_(local)]. The molecular dynamics trajectories described in this work are available at https://www.deshawresearch.com/downloads/download_trajectory_sarscov2.cgi/.
