# Supplementary material for: Structural basis for backtracking by the SARS-CoV-2 replication–transcription complex
Source: Proc Natl Acad Sci U S A. 2021 Apr 21;118(19):e2102516118. doi: 10.1073/pnas.2102516118 (PMC8126829; doi:10.1073/pnas.2102516118)
Supplement: Supplementary File [file pnas.2102516118.sapp.pdf]

PNAS
www.pnas.org

Supplementary Information for

Structural basis for backtracking by the SARS-CoV-2 replication-transcription complex

Brandon Malone[1,8], James Chen[1,8], Qi Wang[2], Eliza Llewellyn[1], Young Joo Choi[1], Paul Dominic B. Olinares[3], Xinyun Cao[4], Carolina Hernandez[5], Edward T. Eng[5], Brian T. Chait[3], David E. Shaw[2,6], Robert Landick[4,7], Seth A. Darst[1,9], Elizabeth A. Campbell[1,9,10]

[1]Laboratory of Molecular Biophysics, The Rockefeller University, New York, NY, 10065 USA.

[2]D. E. Shaw Research, New York, NY 10036 USA.

[3]Laboratory of Mass Spectrometry and Gaseous Ion Chemistry, The Rockefeller University, New York, NY, 10065 USA.

[4]Department of Biochemistry, University of Wisconsin-Madison, Madison, WI 53706 USA.

[5]The National Resource for Automated Molecular Microscopy, Simons Electron Microscopy Center, New York Structural Biology Center, New York, NY, 10027 USA.

[6]Department of Biochemistry and Molecular Biophysics, Columbia University, New York, NY 10032 USA.

[7]Department of Bacteriology, University of Wisconsin-Madison, Madison, WI 53706 USA.

[8]These authors contributed equally: Brandon Malone, James Chen.

[9]Correspondence to: darst@rockefeller.edu, campbee@rockefeller.edu

Supplementary Information Text

METHODS

Structural biology software was accessed through the SBGrid consortium (1).

Protein expression and purification.

SARS-CoV-2 nsp12. SARS-CoV-2 nsp12 was expressed and purified as described (1). A pRSFDuet-1 plasmid expressing SARS-CoV-2 His_6-SUMO-nsp12 (Addgene plasmid 159107) was transformed into *Escherichia coli* (*Eco*) BL21-CodonPlus cells (Agilent). Cells were grown, followed by the addition of isopropyl β-d-1-thiogalactopyranoside (IPTG) to induce protein expression overnight. Cells were collected by centrifugation, resuspended and lysed in a continuous-flow French press (Avestin). The lysate was cleared by centrifugation, loaded onto a HiTrap Heparin HP column (Cytiva), and then eluted using a salt gradient. The fractions containing nsp12 were pooled and loaded onto a HisTrap HP column (Cytiva), washed, and eluted. Eluted nsp12 was dialyzed overnight in the presence of His_6-Ulp1 SUMO protease. Cleaved nsp12 was passed through a HisTrap HP column (Cytiva). Flow-through was collected, concentrated by centrifugal filtration (Amicon), and loaded on a Superdex 200 Hiload 16/600 (Cytiva) for size-exclusion chromatography. Glycerol was added to the purified nsp12, aliquoted, flash frozen with liquid N_2, and stored at -80°C.

SARS-CoV-2 nsp7/8. SARS-CoV-2 nsp7/8 was expressed and purified as described (1). The pCDFDuet-1 plasmid expressing SARS-CoV-2 His_6-ppx-nsp7/8 (ppx is a Prescission Protease cleavage site; Addgene plasmid 159092) was transformed into *Eco* BL21(DE3). Cells were grown and protein expression was induced overnight by the addition of IPTG. Cells were collected by centrifugation, resuspended, and lysed in a continuous-flow French press (Avestin). The lysate was cleared by centrifugation, then loaded onto a HisTrap HP column (Cytiva), washed, and eluted. Eluted nsp7/8 was dialyzed overnight in the presence of His_6-Prescission Protease to cleave the His_6-tag. Cleaved nsp7/8 was passed through a HisTrap HP column (Cytiva). Flow-through was collected,

concentrated by centrifugal filtration (Amicon), and loaded onto a Superdex 75 Hiload 16/600 (Cytiva). Glycerol was added to the purified nsp7/8, aliquoted, flash frozen with liquid N_2, and stored at -80°C.

SARS-CoV-2 nsp13. SARS-CoV-2 nsp13 was expressed and purified as described (1). The pet28 plasmid containing SARS-CoV-2 His_6-ppx-nsp13 (Addgene plasmid 159390) was transformed into *Eco* Rosetta(DE3) (Novagen). Cells were grown, followed by the addition of IPTG to induce protein expression overnight. Cells were collected by centrifugation, resuspended, and lysed in a continuous-flow French press (Avestin). The lysate was cleared by centrifugation, then loaded onto a HisTrap HP column (Cytiva), washed, and eluted. Eluted nsp13 was dialyzed overnight in the presence of His_6-Prescission Protease to cleave His_6-tag. Cleaved nsp13 was passed through a HisTrap HP column (Cytiva). Flow-through was collected, concentrated by centrifugal filtration (Amicon), and loaded onto a Superdex 200 Hiload 16/600 (Cytiva). Glycerol was added to the purified nsp13, aliquoted, flash frozen with liquid N_2, and stored at -80°C.

Native electrophoretic mobility shift assays. Nsp12 or nsp12-D760A were incubated with 3-fold molar excess of nsp7/8 in transcription buffer (120 mM K-acetate, 20 mM HEPES pH 8, 10 mM $MgCl_2$, 2 mM DTT) to assemble holo-RdRp (2 μM final). The resulting complex was incubated with 1 μM of annealed RNA scaffold (Horizon Discovery) for 5 minutes at 30°C. Nsp13 and pre-mixed ADP and AlF_3 (Sigma-Aldrich) were added to a final concentration of 2 μM and 2 mM, respectively, and incubated for an additional 5 minutes at 30°C. Reactions were analyzed by native gel electrophoresis on a 4.5% polyacrylamide native gel (37.5:1 acrylamide:bis-acrylamide) in 1X TBE (89 mM Tris, 89 mM boric acid, 1 mM EDTA) at 4°C. The gel was stained with Gel-Red (Biotium).

Native mass spectrometry (nMS) analysis. The reconstituted sample containing 4 μM RTC and 8 μM nsp13 incubated with 2 mM ADP-AlF_3 was

buffer-exchanged into 150 mM ammonium acetate, 0.01% Tween-20, pH 7.5 using a Zeba microspin desalting column with a 40 kDa MWCO (ThermoFisher Scientific). For nMS analysis, a 2–3 µL aliquot of the buffer-exchanged sample was loaded into a gold-coated quartz capillary tip that was prepared in-house and then electrosprayed into an Exactive Plus with extended mass range (EMR) instrument (Thermo Fisher Scientific) with a static direct infusion nanospray source (2). The MS parameters used: spray voltage, 1.2 kV; capillary temperature, 150 °C; in-source dissociation, 0 V; S-lens RF level, 200; resolving power, 17,500 at *m/z* of 200; AGC target, 1×10^6; maximum injection time, 200 ms; number of microscans, 5; injection flatapole, 6 V; interflatapole, 4 V; bent flatapole, 4 V; high energy collision dissociation (HCD), 200 V; ultrahigh vacuum pressure, 7.2×10^{-10} mbar; total number of scans, at least 100. Mass calibration in positive EMR mode was performed using cesium iodide. For data processing, the acquired MS spectra were visualized using Thermo Xcalibur Qual Browser (v. 4.2.47). MS spectra deconvolution was performed either manually or using the software UniDec v. 4.2.0 (3, 4). The following parameters were used for the UniDec processing: m/z range, 7,000 – 10,000 Th; background subtraction, subtract curved at 100; smooth charge state distribution, enabled; peak shape function, Gaussian; Beta Softmax distribution parameter, 20.

The expected masses for the component proteins based on previous nMS experiments (1) include nsp7: 9,137 Da; nsp8 (N-terminal Met lost): 21,881 Da; nsp13 (post-protease cleavage, has three Zn^{2+} ions coordinated with 9 deprotonated cysteine residues): 67,464 Da, and nsp12 (has two Zn^{2+} ions coordinated with 6 deprotonated cysteine residues): 106,785 Da. The mass of the assembled RNA duplex scaffold is 30,512 Da.

Experimental masses were reported as the average mass ± standard deviation (S.D.) across all the calculated mass values within the observed charge state series. Mass accuracies were calculated as the % difference between the measured and expected masses relative to the expected mass. The observed mass accuracies ranged from 0.016 – 0.035%.

Preparation of SARS-CoV-2 nsp13-BTC$_5$ for Cryo-EM. Purified nsp12 and nsp7/8 were mixed in a 1:3 molar ratio and incubated at 22° C for 15 minutes. The mixture was buffer-exchanged into cryo-EM buffer (20 mM HEPES pH 8.0, 150 mM K-acetate, 10 mM $MgCl_2$, 1 mM DTT) using Zeba desalting columns (ThermoFisher Scientific) and incubated with annealed BTC$_5$-scaffold (Fig. 1A) in a 1:1.5 molar ratio. Purified nsp13 was concentrated by centrifugal filtration (Amicon) and buffer exchanged into cryo-EM buffer using Zeba desalting columns. Buffer exchanged nsp13 was mixed with ADP and AlF_3 and then added to nsp7/8/12/RNA scaffold at a molar ratio of 1:1 with a final concentration of 2 mM ADP-AlF_3. Complex was incubated for 5 minutes at 30° C and further concentrated by centrifugal filtration (Amicon).

Cryo-EM grid preparation. Prior to grid freezing, 3-([3-cholamidopropyl]dimethylammonio)-2-hydroxy-1-propanesulfonate (CHAPSO, Anatrace) was added to the sample (8 mM final), resulting in a final complex concentration of 10 µM. The final buffer condition for the cryo-EM sample was 20 mM HEPES pH 8.0, 150 mM K-acetate, 10 mM $MgCl_2$, 1 mM DTT, 2 mM ADP-AlF_3, 8 mM CHAPSO. C-flat holey carbon grids (CF-1.2/1.3-4Au, Electron Microscopy Sciences) were glow-discharged for 20 s prior to the application of 3.5 µL of sample. Using a Vitrobot Mark IV (ThermoFisher Scientific), grids were blotted and plunge-frozen into liquid ethane with 90% chamber humidity at 4°C.

Cryo-EM data acquisition and processing. Structural biology software was accessed through the SBGrid consortium (5). Grids were imaged using a 300 kV Titan Krios (ThermoFisher Scientific) equipped with a K3 camera (Gatan) and a BioQuantum imaging filter (Gatan). Images were recorded using Leginon (6) with a pixel size of 1.065 Å/px (micrograph dimensions of 5,760 x 4,092 px) over a nominal defocus range of -0.8 µm to -2.5 µm and 30 eV slit width. Movies were recorded in "counting mode" (native K3 camera binning 2) with ~30 e-/px/s in

dose-fractionation mode with subframes of 50 ms over a 2.5 s exposure (50 frames) to give a total dose of ~66 e-/Å. Dose-fractionated movies were gain-normalized, drift-corrected, summed, and dose-weighted using MotionCor2 (7). The contrast transfer function (CTF) was estimated for each summed image using the Patch CTF module in cryoSPARC v2.15.0 (8) Particles were picked and extracted from the dose-weighted images with box size of 256 px using cryoSPARC Blob Picker and Particle Extraction. The entire dataset consisted of 10,685 motion-corrected images with 4,961,691 particles. Particles were sorted using cryoSPARC 2D classification (N=100), resulting in 2,412,034 curated particles. Initial models (Ref 1: decoy 1, Ref 2: complex, Ref 3: decoy 2; SI Appendix; Fig. S2) were generated using cryoSPARC *ab initio* Reconstruction on a subset of 85,398 particles. Particles were further curated using Ref 1-3 as 3D templates for cryoSPARC Heterogeneous Refinement (N=6), resulting in the following: class1 (Ref 1), 258,097 particles; class2 (Ref 1), 263,966 particles; class3 (Ref 2), 668,743 particles; class4 (Ref 2), 665,480 particles; class5 (Ref 3), 280,933 particles; class6 (Ref 3), 274,815 particles. Particles from class3 and class4 were combined and further curated with another round of Heterogeneous Refinement (N=6), resulting in the following: class1 (Ref 1), 67,639 particles; class2 (Ref 1), 61,097 particles; class3 (Ref 2), 553,368 particles; class4 (Ref 2), 554,581 particles; class5 (Ref 3), 42,114 particles; class6 (Ref 3), 55,424 particles. Curated particles from class3 and class4 were combined, re-extracted with a box size of 320 px, and further classified using Ref 2 as a 3D template for cryoSPARC Heterogeneous Refinement (N=4). Classes from this round of Heterogeneous Refinement (N=4) were as follows: class1 (Ref 2), 871,163 particles; class2 (Ref 2), 77,769 particles; class3 (Ref 2), 61,489 particles; class4 (Ref 2), 64,026 particles. Particles from class1 and class2 were combined and further sorted using Heterogeneous Refinement (N=4) using class maps as templates, resulting in the following: class1, 134,536 particles; class2, 270,170 particles; class3, 294,162 particles; class4, 172,295 particles. Classification revealed two unique classes: $nsp13_1$-BTC (class1 and class2) and $nsp13_2$-BTC (class3 and class4). Particles within each class were further processed using

RELION 3.1-beta Bayesian Polishing(9, 10). Polished particles were refined using cryoSPARC Non-uniform Refinement, resulting in structures with the following particle counts and nominal resolutions: nsp13$_1$-BTC (404,706 particles; 3.40 Å) and nsp13$_2$-BTC (466,457 particles; 3.45 Å).

To improve the resolution of the RNA in the BTC, particles from both classes were combined in a cryoSPARC Non-uniform Refinement and density corresponding to nsp13 was subtracted. Subtracted particles were further refined with cryoSPARC Local Refinement using a mask encompassing the BTC and a fulcrum point defined on the backtracked RNA. This map, BTC$_5$(local), contained 871,163 particles with a nominal resolution of 3.23 Å.

To improve the density of nsp13.2 in the nsp13$_2$-BTC map, particles were subtracted using a mask defined around nsp13.2, leaving residual signal for only nsp13.2. Subtracted particles were classified (N=4) in RELION 3.1 beta using a mask around nsp13.2, resulting in the following classes: class1, 71,607 particles; class2, 163,540 particles; class3, 176,461 particles; class4, 54,849 particles. Subtracted particles in class1 and class2 were combined and reverted back to the original particles, followed by refinement using cryoSPARC Non-uniform Refinement. The resulting map of nsp13$_2$-BTC contains 235,147 particles with nominal resolution of 3.59 Å. Local resolution calculations were generated using blocres and blocfilt from the Bsoft package (11).

Model building and refinement. Initial models were derived from PDB: 6XEZ (1). The models were manually fit into the cryo-EM density maps using Chimera (12) and rigid-body and real-space refined using Phenix real_space_refine (13). For real-space refinement, rigid body refinement was followed by all-atom and B-factor refinement with Ramachandran and secondary structure restraints. Models were inspected and modified in Coot (14).

4-thiouridine crosslinking. Nsp12 or nsp12-D760A were incubated with 3-fold molar excess of nsp7/8 to assemble holo-RdRp (2 μM final) in transcription buffer. The resulting holo-RdRp was added to a modified RNA scaffold (SI Appendix; Fig. S5A) containing a photoactivable 4-thiouridine base (Horizon Discovery) which was 5'-labelled by T4-polynucleotide kinase (New England Biolabs) with γ-^{32}P-ATP (Perkin-Elmer). The holo-RdRp/RNA complex was left to incubate for 5 minutes at 30°C in the dark. Nsp13 and ATP were added to a final concentration of 2 uM and 2 mM, respectively, and incubated for five minutes at 30°C in the dark. The reaction mixture was transferred to a Parafilm covered aluminum block at 4°C and irradiated with a 365-nm handheld UV lamp. Reactions were quenched with LDS sample loading buffer (ThermoFisher Scientific) and analyzed by gel electrophoresis on a NuPAGE 4-12% Bis-Tris gel (ThermoFisher) at 150 Volts for 1 hour and visualized by autoradioagraphy.

Molecular dynamics simulations

General simulation setup and parameterization. Proteins, ADP, and ions were parameterized with the DES-Amber SF1.0 force field (15). RNAs were parameterized with the Amber ff14 RNA force field (16) with modified electrostatic, van der Waals, and torsional parameters to more accurately reproduce the energetics of nucleobase stacking (17). The systems were solvated with water parameterized with the TIP4P-D water model (18) and neutralized with 150 mM NaCl buffer. The systems each contained ~887,000 atoms in a 190×190×190 Å cubic box.

Systems were first equilibrated on GPU Desmond using a mixed NVT/NPT schedule (19), followed by a 1 μs relaxation simulation on Anton, a special-purpose machine for molecular dynamics simulations (20). All production simulations were performed on Anton and initiated from the last frame of the relaxation simulation. Production simulations were performed in the NPT ensemble at 310 K using the Martyna-Tobias-Klein barostat (21). The simulation time step was 2.5 fs, and a modified r-RESPA integrator (22, 23) was used in

which long-range electrostatic interactions were evaluated every three time steps. Electrostatic forces were calculated using the *u*-series method (24). A 9-Å cutoff was applied for the van der Waals calculations.

System preparation. The nsp13$_2$-BTC$_{-1U+1C}$ and the nsp13$_2$-BTC$_{-1U+1U}$ complexes were prepared from the cryo-EM structure of the nsp13$_2$-BTC$_5$. AlF_3 and CHAPSO were removed. Cytosines at the +2 and +3 positions of the p-RNA were removed, and the cytosine at −1 was mutated to uracil. The resulting p-RNA had a matched −1U and a mismatched +1C in nsp13$_2$-BTC$_{-1U+1C}$, and a matched −1U and +1U in nsp13$_2$-BTC$_{-1U+1U}$. Missing loops and termini in proteins were capped with ACE/NME capping groups. Missing RNA nucleotides (chain T +7 to +3) were manually added. The two complexes were prepared for simulation using the Protein Preparation Wizard in Schrödinger Maestro. After a 1 µs relaxation simulation of the nsp13$_2$-BTC$_{-1U+1C}$ complex, the −1U of the p-RNA formed a Watson-Crick base pair with the −1A in the t-RNA, and the +1C of p-RNA formed a non-Watson-Crick C-A hydrogen bond with the +1A of the t-RNA in the active site. After a 1 µs relaxation simulation of the nsp13$_2$-BTC$_{-1U+1U}$ complex, the −1U and +1U of the p-RNA formed Watson-Crick base pairs with the −1A and +1A of the t-RNA respectively.

Simulation analysis. All simulations were visually inspected using the in-house visualization software Firefly. The average root-mean-square deviation (RMSD) was calculated for +1C (or +1U) of the p-RNA between the last frame of the 1 µs relaxation simulation and instantaneous structures from the trajectories, aligned on the entire nps12 module.

Quantification and statistical analysis. The nMS spectra were visualized using Thermo Xcalibur Qual Browser (versions 3.0.63 and 4.2.27), deconvolved using UniDec versions 3.2 and 4.1 (3, 4) and plotted using the m/z software (Proteometrics LLC, New York, NY). Experimental masses (SI Appendix; Fig. S1B and C) were reported as the average mass ± standard deviation across

all the calculated mass values obtained within the observed charge state distribution.

The local resolution of the cryo-EM maps (SI Appendix; Fig. S3B-D) was estimated using blocres (11) with the following parameters: box size 15, sampling 1.1, and cutoff 0.5. Directional 3D FSC (SI Appendix; Fig. S3H-J) were calculated by 3DFSC (25). The quantification and statistical analyses for model refinement and validation were generated using MolProbity (26) and PHENIX (13).

Table S1. Cryo-EM data collection, refinement, and validation statistics.

	**nsp7/8/12/13/BTC_scaffold/ADP-AlF_3/CHAPSO**		
**Sample ID**	$nsp13_1$-BTC	$nsp13_2$-BTC	BTC (local)
**EMDB**	EMD-23007	EMD-23008	EMD-23009
**PDB**	7KRN	7KRO	7KRP
**Data collection and processing**			
Microscope		TFS Titan Krios	
Voltage (kV)		300	
Detector		Gatan K3 Camera	
Electron exposure (e–/Å2)		66	
Defocus range (μm)		-0.8 to -2.5	
Data collection mode		Counting Mode	
Nominal Magnification		81,000x	
Pixel size (Å)		1.065	
Symmetry imposed		C1	
Initial particle images (no.)		4,961,691	
Final particle images (no.)	404,706	235,147	871,163
Map resolution (Å) - FSC threshold 0.143	3.40	3.59	3.23
Map resolution range (Å)	2.5-5.0	2.5-5.0	2.5-5.1
**Refinement**			
Initial model used (PDB code)	6XEZ	6XEZ	6XEZ
Map sharpening B factor (Å2)	-139.6	-127.6	-103.9

Model composition			
Non-hydrogen atoms	17351	21988	12561
Protein residues	1963	2553	1373
Nucleic acid residues	80	80	73
Ligands	5 Zn^{2+}, 2 Mg^{2+}, 3 CHAPSO, 2 ADP, 1 AlF_3	8 Zn^{2+}, 3 Mg^{2+}, 3 CHAPSO, 3 ADP, 2 AlF_3	2 Zn^{2+}, 1 Mg^{2+}, 3 CHAPSO, 1 ADP
B factors (Å2)			
Protein	45.65	74.56	38.31
Nucleic acid	128.79	163.6	140.77
Ligands	59.03	78.99	46.55
R.m.s. deviations			
Bond lengths (Å)	0.007	0.007	0.004
Bond angles (°)	0.711	0.735	0.609
Validation			
MolProbity score	2.66	2.68	1.97
Clashscore	9.18	8.96	6.12
Poor rotamers (%)	7.54	9.4	4.18
Ramachandran plot			
Favored (%)	91.4	92.76	97.07
Allowed (%)	8.6	6.93	2.93
Disallowed (%)	0	0.31	0

Table S2. Comparison of $nsp13_1$-BTC_5 and $nsp13_2$-BTC_5 structures.

	PyMOL align command over all α-carbons[a]	PyMOL align command over nsp12 α-carbons[b]	
root-mean-square deviation (Å) (# of α-carbons included in alignment)	0.459 (1336)	0.214 (853)	
subunit	rms_cur	rms_cur	
nsp12	0.298 (927)	0.278 (927)	
nsp8a	1.288 (186)	1.411 (186)	N-term ext. (residues 1-98): 1.965
			residues 99-186: 0.345
nsp7	0.333 (75)	0.337 (75)	
nsp8b	0.841 (185)	0.912 (185)	N-term ext. (residues 1-82): 1.116
			residues 83-186: 0.737
nsp13.1	8.378 (590)	8.332 (590)	

[a]PyMOL command: align ($nsp13_1$-BTC_5 and name CA),($nsp13_2$-BTC_5 and name CA)

[b]PyMOL command: align ($nsp13_1$-BTC_5 and chain A and name CA),($nsp13_2$-BTC_5 and chain A and name CA)

SUPPLEMENTAL FIGURES

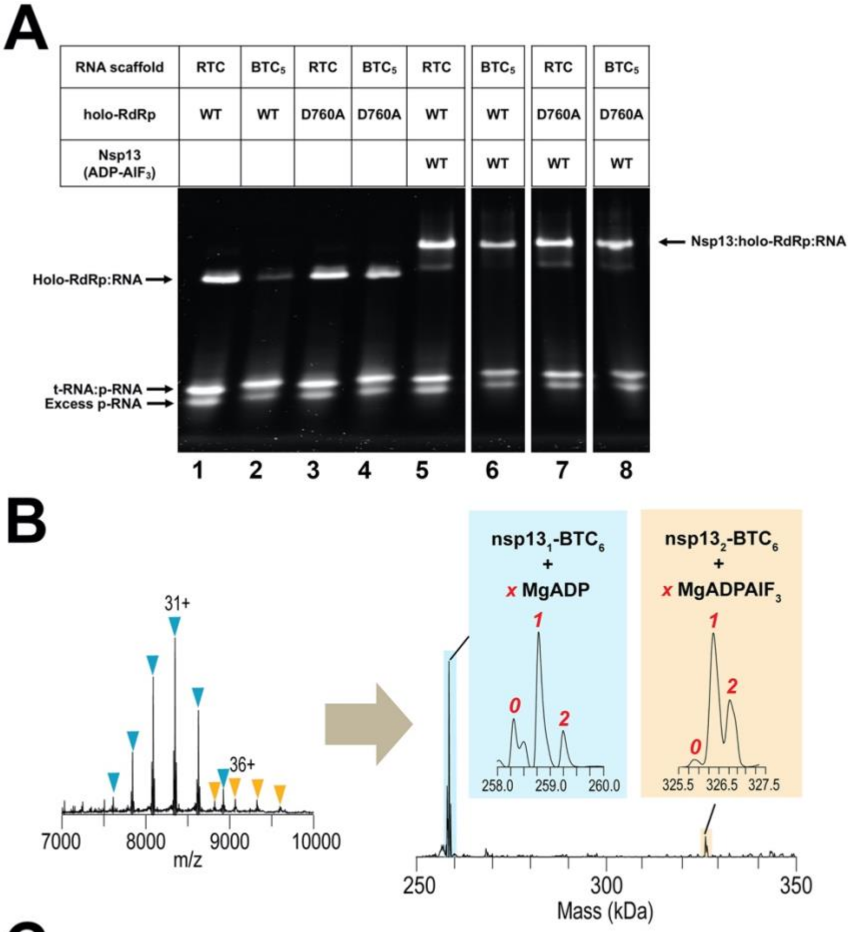

C

Mass assignments from nMS analysis of BTC_6 incubated with nsp13 and $MgADPAlF_3$

Protein Assembly	Measured Mass ± SD (Da)*	Expected Mass (Da)	Δ Mass (Da)	% Mass Error
$nsp13_1$-BTC_6	258,307 ± 2	258,264	42	0.02
$nsp13_1$-BTC_6 + 1 MgADP	258,782 ± 3	258,716	66	0.03
$nsp13_1$-BTC_6 + 2 MgADP	259,246 ± 4	259,167	78	0.03
$nsp13_2$-BTC_6	325,844 ± 34	325,729	116	0.04
$nsp13_2$-BTC_6 + 1 $MgADPAlF_3$	326,342 ± 18	326,264	78	0.02
$nsp13_2$-BTC_6 + 2 $MgADPAlF_3$	326,776 ± 17	326,716	60	0.02

* Calculated from the average and S.D. of all the measured masses across the charge-state distribution ($n \geq 4$).

Fig. S1. Native gel electrophoresis mobility shift assay and nMS analysis of the BTC.

A. A native gel electrophoretic mobility shift assay reveals that wt-holo-RdRp requires nsp13(ADP-AlF_3) to bind the BTC_5-scaffold efficiently (compare lanes 1, 2, and 6) but holo-RdRp with nsp12-D760A does not require nsp13 (lane 4).

B. The nMS spectrum and the deconvolved mass spectrum showing assembly of stable nsp13-BTC_6, formed on a scaffold identical to the BTC_5-scaffold except with six mismatched C's at the p-RNA 3'-end. The peak for the $nsp13_2$-BTC_6 assembly is present at about ~9% intensity relative to the predominant peak from $nsp13_1$-BTC_6. While most of the biochemical and structural analyses were done using the BTC_5-scaffold, we do not expect major differences in the behavior of BTC_6 compared to BTC_5.

C. Mass assignments of the deconvolved peaks from the nMS analysis.

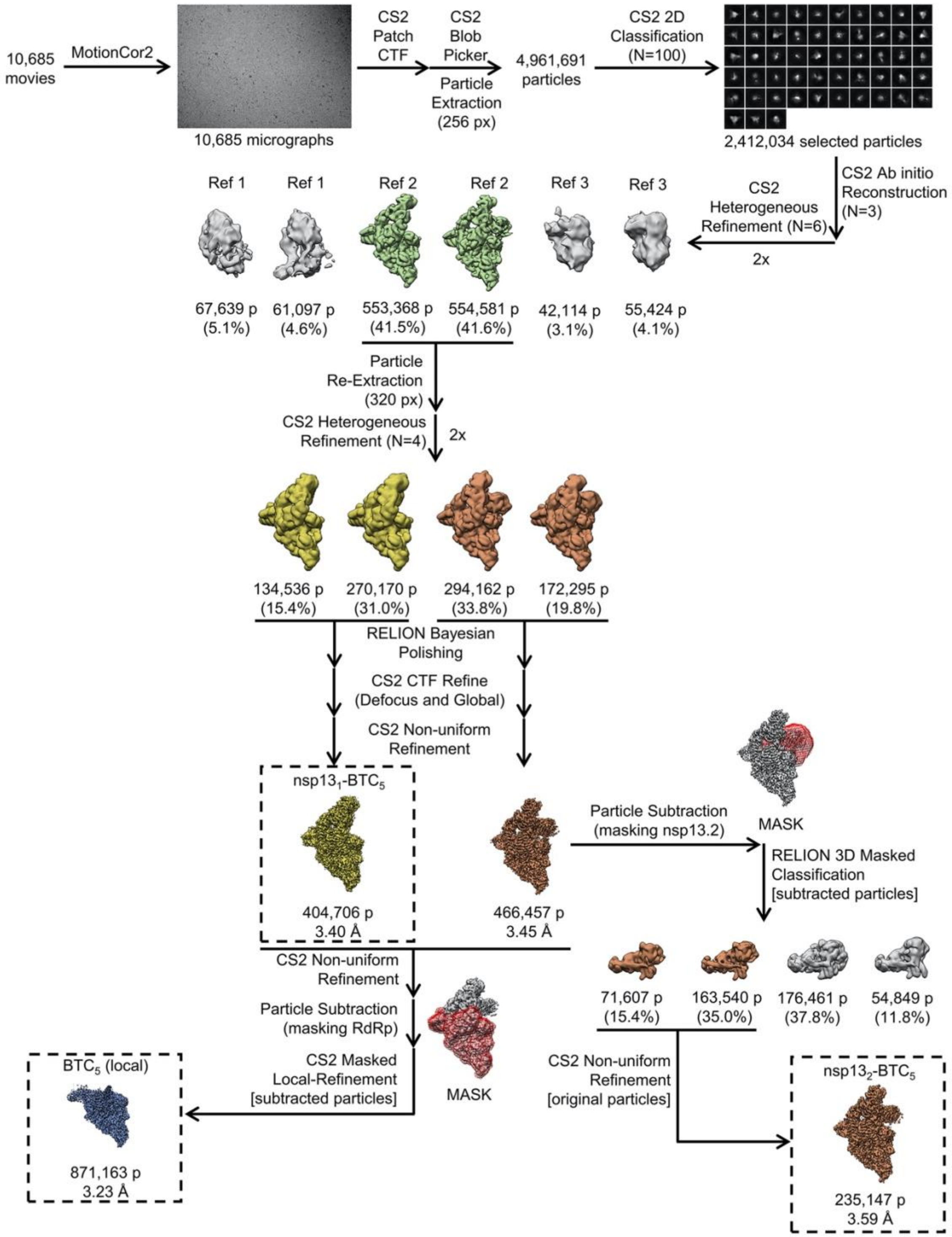

Fig. S2. Cryo-EM processing pipeline.

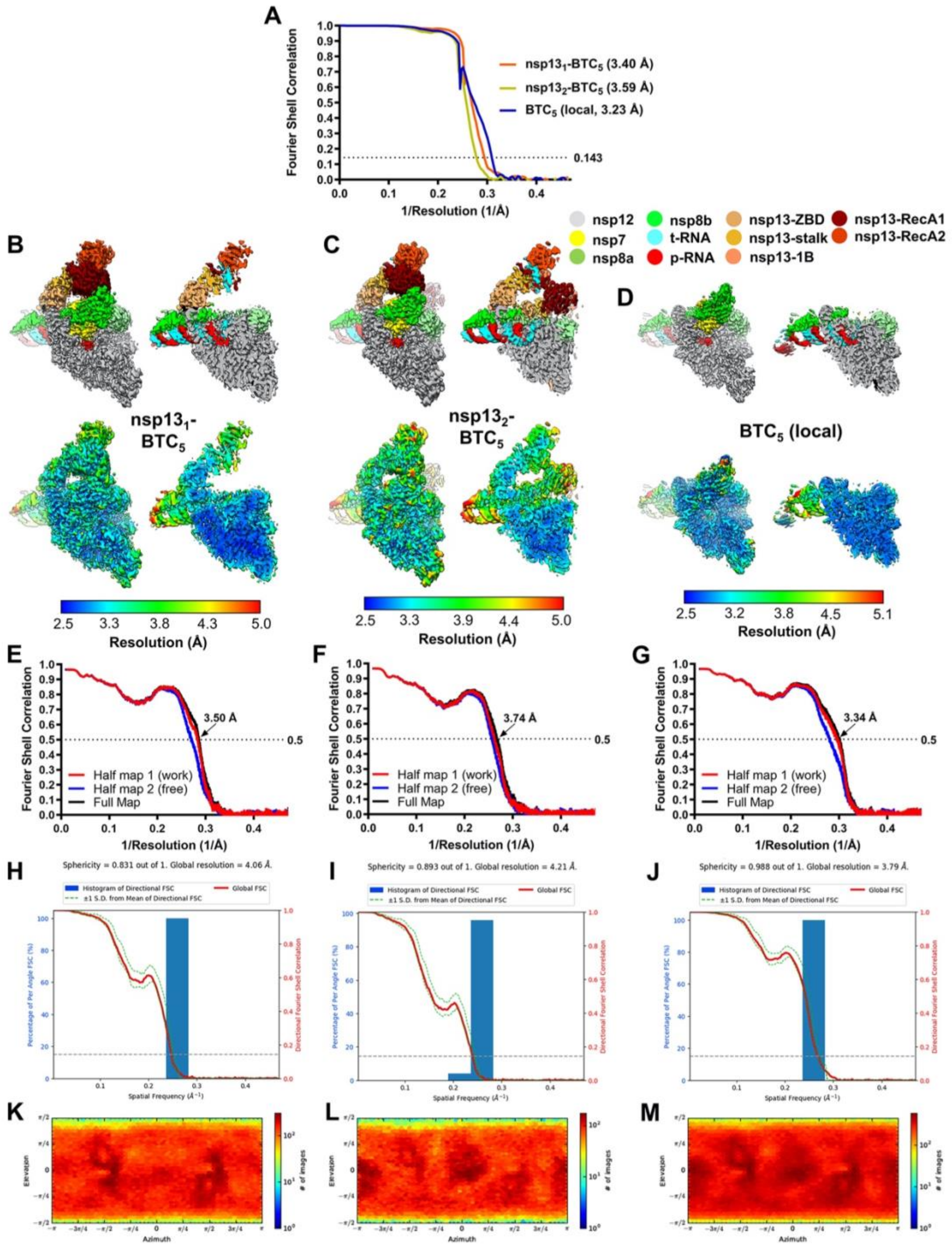

Fig. S3. Cryo-EM analysis.

A. Gold-standard FSC plots for nsp13_1-BTC$_5$, nsp13_2-BTC$_5$, and BTC$_5$(local), calculated by comparing two independently determined half-maps from cryoSPARC (8). The dotted line represents the 0.143 FSC cutoff.

B-D. Cryo-EM reconstructions filtered by local resolution(11). The view on the right is a cross-section.

(top) Colored by subunit according to the color key.

(bottom) Color by local resolution (key on the bottom).

B. $Nsp13_1$-BTC_5.

C. $Nsp13_2$-BTC_5.

D. BTC_5(local).

E – G. FSC calculated between the refined structures and the half map used for refinement (work, red), the other half map (free, blue), and the full map (black).

E. $Nsp13_1$-BTC_5.

F. $Nsp13_2$-BTC_5.

G. BTC_5(local).

H - J, Directional 3D Fourier shell correlation plots, calculated by 3DFSC(25).

H. $Nsp13_1$-BTC_5.

I. $Nsp13_2$-BTC_5.

J. BTC_5(local).

K – M. Particle angular distribution plots calculated in cryoSPARC. Scale shows the number of particles assigned to a particular angular bin. Blue, a low number of particles; red, a high number of particles.

K. $Nsp13_1$-BTC_5.

L. $Nsp13_2$-BTC_5.

M. BTC_5(local).

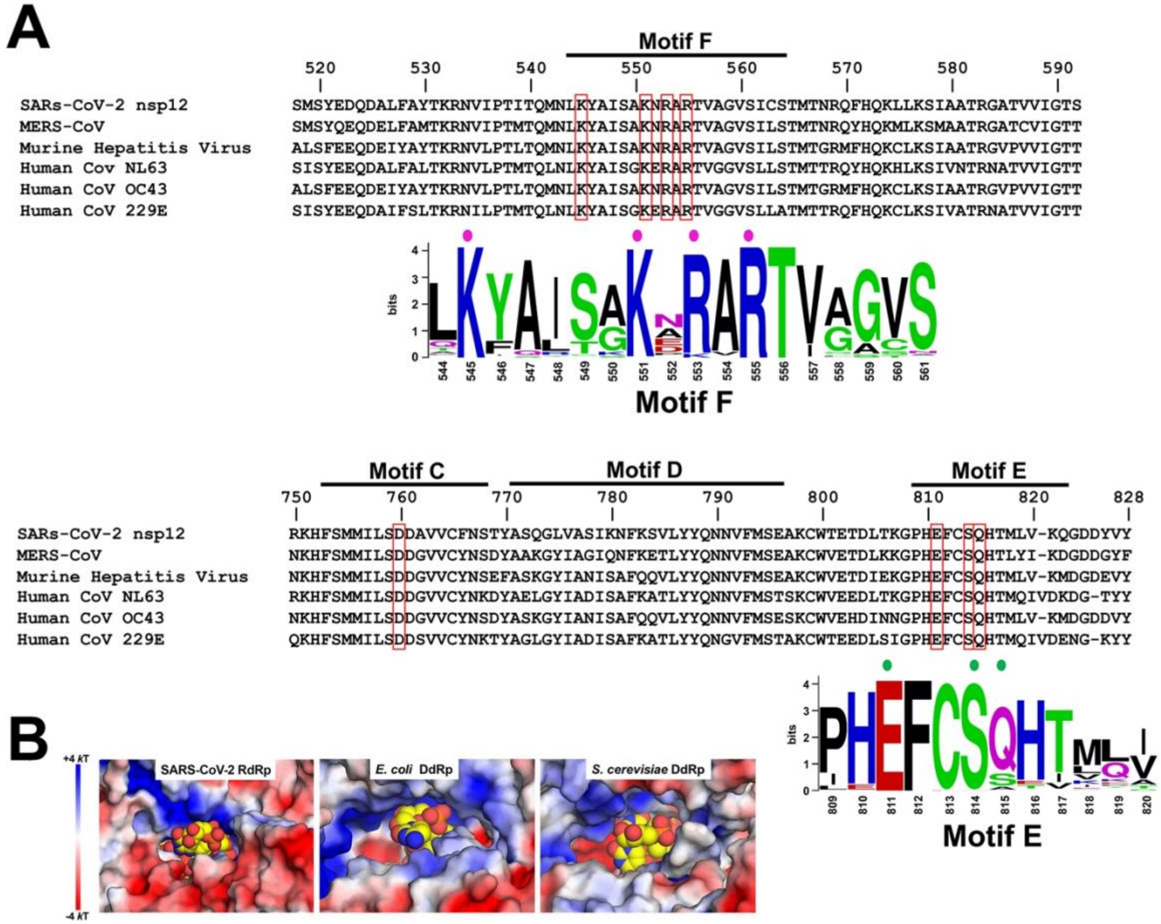

Fig. S4. Sequence conservation of nsp12 homologs and NTP-entry tunnel environment.

A. Sequence alignment of nsp12 homologs from six pathogenic and model CoV family members, covering RdRp motifs (27) (motifs F, C, D, and E denoted at the top of the sequence alignment) architecturally important for the NTP-entry tunnel. Selected residues discussed in the text are highlighted (red outlines). Sequence logos(28) for motif F and motif E are shown, with residues that interact with the backtracked RNA highlighted (colored dots above; see Figure 4). The sequence logos were generated from an alignment of 97 RdRp sequences from α-, β-, γ-, and δ-CoVs (Data S1).

B. Views from the outside into the NTP-entry tunnels of the SARS-CoV-2 BTC (*left*), an *E. coli* DdRp BTC [PDB ID: 6RI9; (29)] and an *S. cerevisiae* DdRp BTC [PDB ID: 3GTP; (30)]. Protein surfaces are colored by the electrostatic surface

potential [calculated using APBS; (31)]. Backtracked RNA is shown as atomic spheres with yellow carbon atoms.

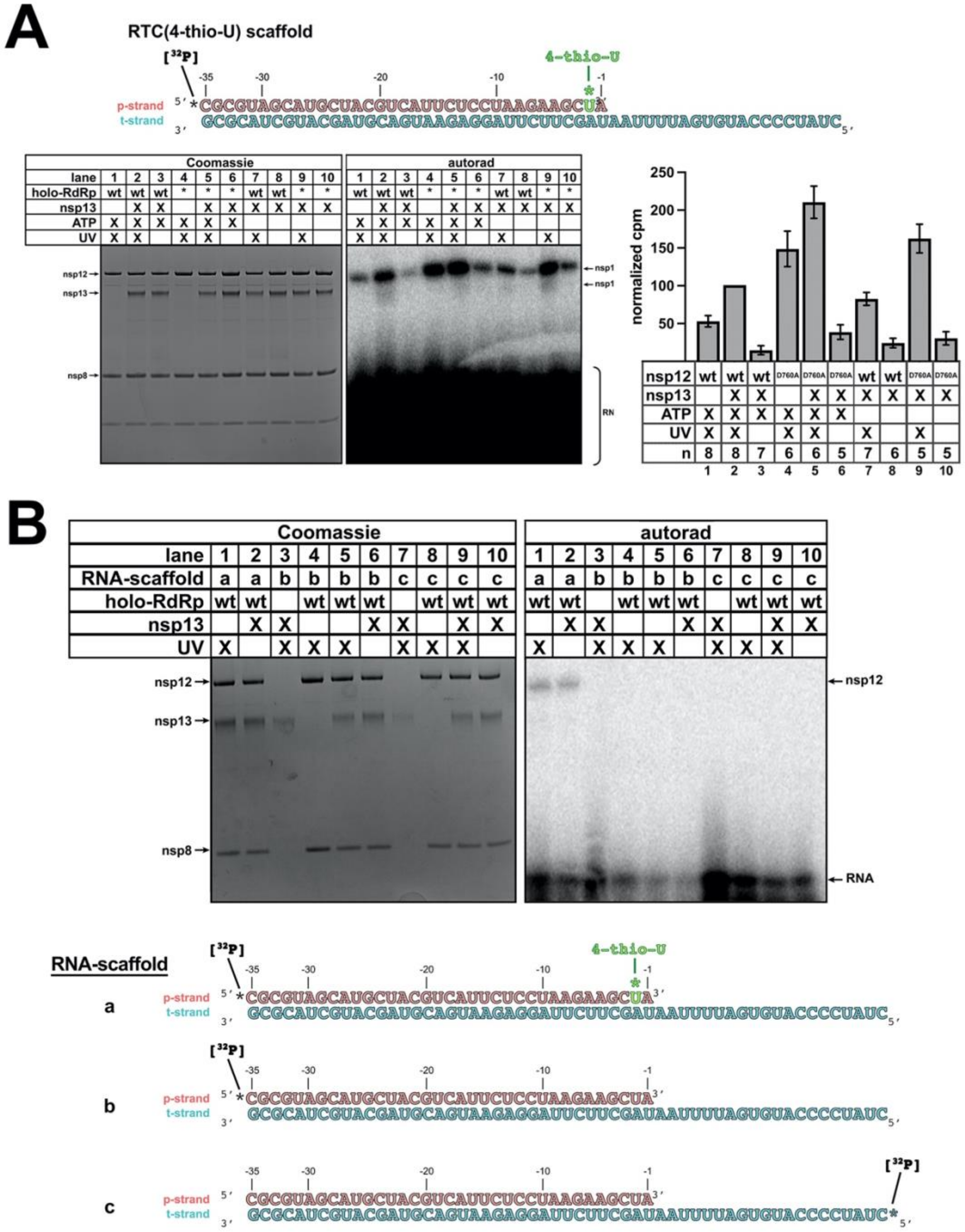

Fig. S5. 4-thio-U crosslinking analysis.

A. Protein-RNA crosslinking analysis: The 5'-[^{32}P]-labelled RTC(4-thio-U)-scaffold and the indicated proteins were incubated along with 2 mM ATP (when present), exposed to UV as indicated, then analyzed by SDS polyacrylamide gel electrophoresis and autoradiography. The positions of nsp8, nsp12, and nsp13 bands are indicated. Holo-RdRp(*) denotes the nsp12-D760A substitution that

facilitates backtracking (see Figure S1A). The two panels show the same SDS polyacrylamide gel (left gel, Coomassie stained; right gel, visualized by autoradiography). The histogram at the far right shows the results of quantitation of the band corresponding to nsp12 by phosphorimagery using ImageJ (32). The error bars denote standard error of the mean (SEM) based on the number of replicates denoted by 'n'. The results between experiments were normalized by equalizing the counts of lane 2 (hence no error bar is shown).

B. Protein-RNA crosslinks are specific. Lanes 1, 2; Analysis using the 5'-[^{32}P]-RTC(4-thio-U)-scaffold (RNA-scaffold 'a' shown on the bottom). Crosslinking to nsp12 serves as a positive control for the crosslinking reaction. Lanes 3-6; Analysis using RNA-scaffold 'b' (RTC-scaffold with 5'-[^{32}P]-labelled p-RNA). Lanes 7-10: Analysis using RNA-scaffold 'c' (RTC-scaffold with 5'-[^{32}P]-labelled t-RNA). The complete absence of protein-RNA crosslinks in lanes 3-10 indicates that the observed protein-RNA crosslinks arise from the 4-thio-U site-specifically incorporated in the p-RNA of the RTC(4-thio-U)-scaffold.

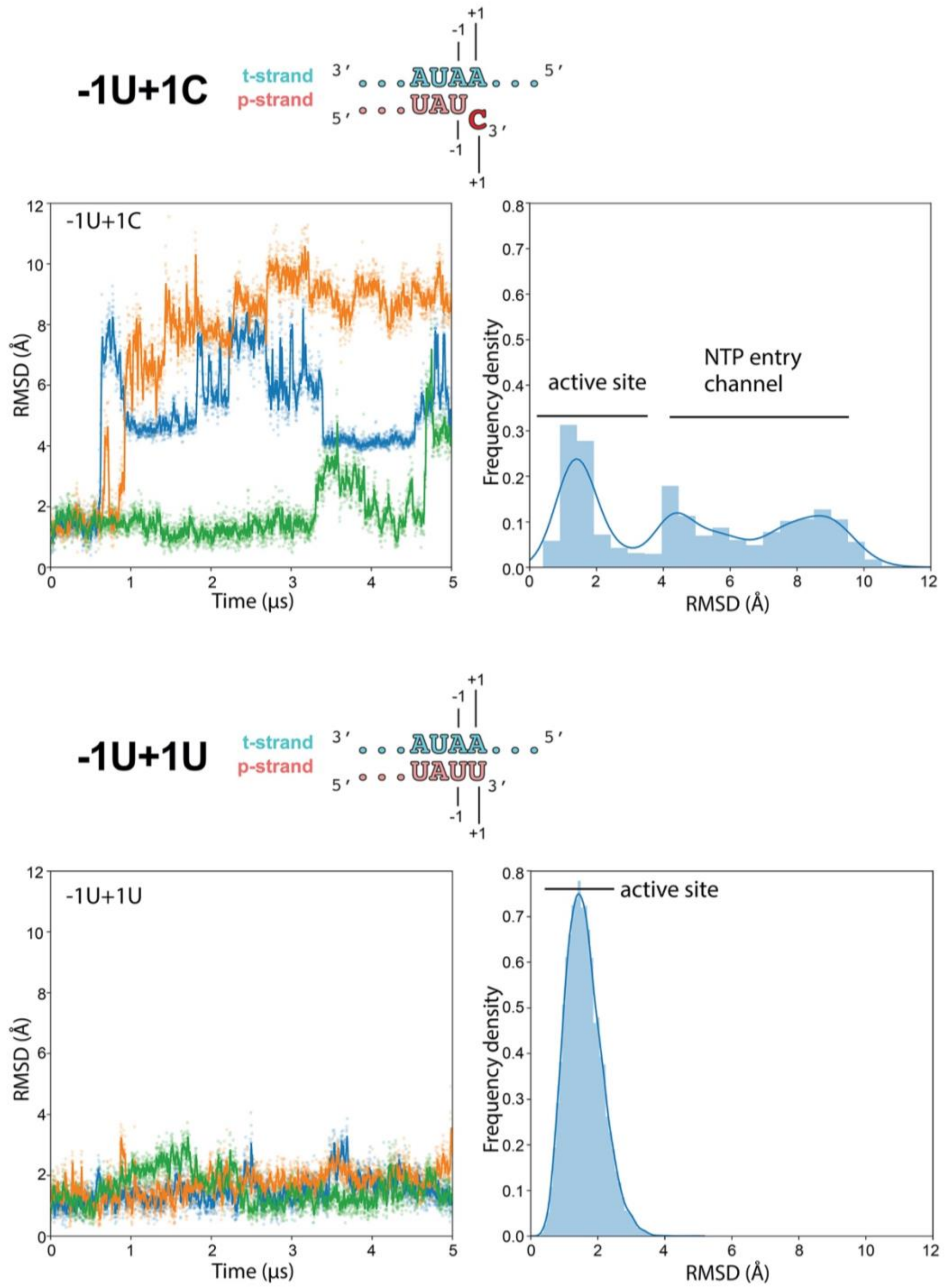

Fig. S6. Molecular dynamics simulations of nsp13$_2$-BTC$_{1U+1C}$ vs. nsp13$_2$-BTC$_{-1U+1U}$.

Molecular dynamics simulations of the nsp13$_2$-BTC$_{-1U+1C}$ (*top*) and nsp13$_2$-BTC$_{-1U+1U}$ (*bottom*) complexes. The complexes were simulated with 3 replicates (green, blue, and orange traces). The schematics illustrate the active-site

proximal nucleotides in each modeled complex. Each complex was simulated with 3 replicates. RMSD values plotted as a function of time represent the heavy-atom RMSD of the +1 nucleotide of the p-RNA (+1C for nsp13$_2$-BTC$_{-1U+1C}$ or matched +1U for nsp13$_2$-BTC$_{-1U+1U}$) compared with the starting configuration (see Methods). The RMSD histograms (plotted on the right) are aggregates of all 3 replicates.

(*top*) Nsp13$_2$-BTC$_{-1U+1C}$. As shown in Figure 5C, the mismatched p-RNA +1C spends about 60% of the time frayed from the t-RNA +1A and near or in the NTP-entry tunnel (RMSD ≥ ~3.5 Å).

(*bottom*) Nsp13$_2$-BTC$_{-1U+1U}$. With the p-RNA +1U matched with the t-RNA +1A for Watson-Crick base pairing, the p-RNA +1U does not fray and spends all of its time in the vicinity of the RdRp active site and base paired with the t-RNA.

SUPPLEMENTAL DATA FILES

Data File S1. Sequence alignment (Clustal format) of α- and β-CoV nsp12 sequences.

SI References

1. J. Chen, *et al.*, Structural basis for helicase-polymerase coupling in the SARS-CoV-2 replication-transcription complex. *Cell* **182**, 1560-1573.e13 (2020).

2. P. D. B. Olinares, B. T. Chait, Methods in Molecular Biology. *Methods Mol Biology Clifton N J* **2062**, 357–382 (2019).

3. D. J. Reid, *et al.*, High-Throughput Deconvolution of Native Mass Spectra. *J Am Soc Mass Spectrom* **30**, 118–127 (2019).

4. M. T. Marty, *et al.*, Bayesian deconvolution of mass and ion mobility spectra: from binary interactions to polydisperse ensembles. *Analytical chemistry* **87**, 4370–4376 (2015).

5. A. Morin, *et al.*, Collaboration gets the most out of software. *eLife* **2**, e01456 (2013).

6. C. Suloway, *et al.*, Automated molecular microscopy: the new Leginon system. *Journal of structural biology* **151**, 41–60 (2005).

7. S. Q. Zheng, *et al.*, MotionCor2: anisotropic correction of beam-induced motion for improved cryo-electron microscopy. *Nature methods* **14**, 331–332 (2017).

8. A. Punjani, J. L. Rubinstein, D. J. Fleet, M. A. Brubaker, cryoSPARC: algorithms for rapid unsupervised cryo-EM structure determination. *Nat Methods* **14**, 290–296 (2017).

9. S. H. W. Scheres, RELION: implementation of a Bayesian approach to cryo-EM structure determination. *Journal of structural biology* **180**, 519–530 (2012).

10. J. Zivanov, *et al.*, New tools for automated high-resolution cryo-EM structure determination in RELION-3. *eLife* **7** (2018).

11. G. Cardone, J. B. Heymann, A. C. Steven, One number does not fit all: mapping local variations in resolution in cryo-EM reconstructions. *Journal of structural biology* **184**, 226–236 (2013).

12. E. F. Pettersen, *et al.*, UCSF Chimera--a visualization system for exploratory research and analysis. *Journal of computational chemistry* **25**, 1605–1612 (2004).

13. P. D. Adams, *et al.*, PHENIX: a comprehensive Python-based system for macromolecular structure solution. *Acta Crystallographica Section D Biological Crystallography* **66**, 213–221 (2010).

14. P. Emsley, K. Cowtan, Coot: model-building tools for molecular graphics. *Acta Crystallographica Section D Biological Crystallography* **60**, 2126–2132 (2004).

15. S. Piana, P. Robustelli, D. Tan, S. Chen, D. E. Shaw, Development of a Force Field for the Simulation of Single-Chain Proteins and Protein–Protein Complexes. *J Chem Theory Comput* **16**, 2494–2507 (2020).

16. J. A. Maier, *et al.*, ff14SB: Improving the Accuracy of Protein Side Chain and Backbone Parameters from ff99SB. *J Chem Theory Comput* **11**, 3696–3713.

17. D. Tan, S. Piana, R. M. Dirks, D. E. Shaw, RNA force field with accuracy comparable to state-of-the-art protein force fields. *Proc National Acad Sci* **115**, 201713027 (2018).

18. S. Piana, A. G. Donchev, P. Robustelli, D. E. Shaw, Water Dispersion Interactions Strongly Influence Simulated Structural Properties of Disordered Protein States. *J Phys Chem B* **119**, 5113–5123 (2015).

19. K. J. Bowers, *et al.*, Scalable Algorithms for Molecular Dynamics Simulations on Commodity Clusters. *Acm Ieee Sc 2006 Conf Sc'06*, 43–43 (2006).

20. D. E. Shaw, *et al.*, Anton 2: Raising the Bar for Performance and Programmability in a Special-Purpose Molecular Dynamics Supercomputer. *Sc14 Int Conf High Perform Comput Netw Storage Analysis*, 41–53 (2014).

21. G. J. Martyna, D. J. Tobias, M. L. Klein, Constant pressure molecular dynamics algorithms. *J Chem Phys* **101**, 4177–4189 (1994).

22. C. Predescu, *et al.*, Computationally efficient molecular dynamics integrators with improved sampling accuracy. *Mol Phys* **110**, 967–983 (2012).

23. M. Tuckerman, B. J. Berne, G. J. Martyna, Reversible multiple time scale molecular dynamics. *J Chem Phys* **97**, 1990–2001 (1992).

24. C. Predescu, *et al.*, The u -series: A separable decomposition for electrostatics computation with improved accuracy. *J Chem Phys* **152**, 084113 (2020).

25. Y. Z. Tan, *et al.*, Addressing preferred specimen orientation in single-particle cryo-EM through tilting. *Nature methods* **14**, 793–796 (2017).

26. V. B. Chen, *et al.*, MolProbity: all-atom structure validation for macromolecular crystallography. *Acta Crystallographica Section D Biological Crystallography* **66**, 12–21 (2010).

27. A. J. W. te Velthuis, Common and unique features of viral RNA-dependent polymerases. *Cell Mol Life Sci Cmls* **71**, 4403–20 (2014).

28. T. D. Schneider, R. M. Stephens, Sequence logos: a new way to display consensus sequences. *Nucleic Acids Research* **18**, 6097–6100 (1990).

29. M. Abdelkareem, *et al.*, Structural Basis of Transcription: RNA Polymerase Backtracking and Its Reactivation. *Mol Cell* **75**, 298-309.e4 (2019).

30. D. Wang, *et al.*, Structural basis of transcription: backtracked RNA polymerase II at 3.4 angstrom resolution. *Science* **324**, 1203–1206 (2009).

31. E. Jurrus, *et al.*, Improvements to the APBS biomolecular solvation software suite. *Protein Sci* **27**, 112–128 (2018).

32. C. A. Schneider, W. S. Rasband, K. W. Eliceiri, NIH Image to ImageJ: 25 years of image analysis. *Nature methods* **9**, 671–675 (2012).
